# Serum IL-2 levels are associated with disease activity and related to dyslipidaemia and the immunological profile in systemic lupus erythematosus

**DOI:** 10.1136/lupus-2025-001870

**Published:** 2026-03-12

**Authors:** Miguel Ángel Gonzalez-Gay, Fuensanta Gómez-Bernal, Beatriz Tejera-Segura, Juan Carlos Quevedo-Abeledo, Enrique García-Barrera, Luisa María Villar, J Gonzalo Ocejo-Vinyals, Raquel Largo, Iván Ferraz-Amaro

**Affiliations:** 1 Medicine, University of Cantabria Faculty of Medicine, Santander, Spain; 2 Rheumatology, Jimenez Diaz Foundation University Hospital, Madrid, Spain; 3 Central Laboratory, Hospital Universitario de Canarias, La Laguna, Spain; 4 Rheumatology, Hospital Universitario Insular de Gran Canaria, Las Palmas de Gran Canaria, Spain; 5 Rheumatology, Hospital Universitario Insulsar Gran Canaria Doctor Negrin, Las Palmas de Gran Canaria, Spain; 6 Immunology, Hospital Universitario Ramon y Cajal, Madrid, Spain; 7 Immunology, Marquis of Valdecilla University Hospital, Santander, Spain; 8 Joint and Bone Research Unit, Rheumatology Department, IIS-Fundacion Jimenez Diaz, Madrid, Spain; 9 Rheumatology, Hospital Universitario de Canarias, La Laguna, Spain; 10 Instituto de Investigaciones Sanitarias de Canarias (IISC), Tenerife, Spain; 11 Department of Internal Medicine, University of La Laguna (ULL), Tenerife, Spain

**Keywords:** Lupus Erythematosus, Systemic, Cardiovascular Diseases, Inflammation, Autoimmune Diseases, Autoimmunity

## Abstract

**Introduction:**

Systemic lupus erythematosus (SLE) is an autoimmune disorder characterised by multisystem involvement, frequently including cardiovascular manifestations. Interleukin 2 (IL-2), a key cytokine in immune regulation, plays a pivotal role in maintaining tolerance by promoting regulatory T cell function. The relationship between serum IL-2 levels and specific SLE features remains incompletely defined. The aim of our study was to investigate the associations between serum IL-2 concentrations and disease activity, inflammatory markers, autoantibody profiles as well as cardiovascular and metabolic parameters in a well-characterised cohort of patients with SLE.

**Methods:**

In this cross-sectional study, 235 patients with SLE were recruited and characterised, including assessment of autoantibody profiles, disease activity indices (SLE Disease Activity Index-2000 (SLEDAI-2K)), Damage Index and remission status. Cardiovascular characteristics were evaluated, encompassing lipid profiles, insulin resistance indices and carotid ultrasound parameters such as plaque presence, intima-media thickness and arterial stiffness. Serum IL-2 concentrations were quantified using an ultrasensitive technique, the Single Molecule Array (Simoa). Multivariable linear regression was conducted to examine the associations between circulating IL-2 levels and clinical as well as cardiovascular manifestations of SLE.

**Results:**

In multivariable analyses, serum IL-2 levels showed significant positive associations with inflammatory markers including C reactive protein and IL-6. Disease activity, assessed by SLEDAI-2K, was positively correlated with IL-2 levels. Positive and significant associations were also noted, after adjustment for covariates, between IL-2 and specific autoantibodies, including anti-DNA, anti-Sjögren’s syndrome antigen A (SSA), anti-Sjögren’s syndrome antigen B (SSB), anti-Smith (Sm) and antiribosome. Regarding cardiovascular disease factors, triglycerides were positively associated with IL-2 whereas high-density lipoprotein-cholesterol exhibited an inverse relationship.

**Conclusion:**

Significant correlations exist between serum IL-2 levels and markers of inflammation, lipid profile, disease activity and specific autoantibody profiles in patients with SLE. These findings underscore the pivotal role of IL-2 in SLE immunopathogenesis, reflecting its intricate involvement in modulating both inflammatory responses and metabolic pathways.

**WHAT IS ALREADY KNOWN ON THIS TOPIC**

- SLE is a complex autoimmune disease with multi-organ involvement, including significant cardiovascular risk.
- Interleukin 2 (IL-2) plays a critical role in immune regulation, but the detailed associations between serum IL-2 levels and specific clinical, immunological and metabolic features in SLE have not been fully elucidated.

**WHAT THIS STUDY ADDS**

- Our study demonstrates significant correlations between serum IL-2 and disease activity, inflammatory markers, autoantibody profiles and lipid metabolism parameters in patients with SLE, providing novel insights into the immunometabolic landscape of this disease.

**HOW THIS STUDY MIGHT AFFECT RESEARCH, PRACTICE OR POLICY**

- These findings highlight the potential of IL-2 as a biomarker for monitoring disease activity and cardiovascular risk in SLE, paving the way for improved management strategies and targeted therapies aimed at reducing morbidity in this patient population.

## Introduction

 Systemic lupus erythematosus (SLE) is a chronic autoimmune disorder of unknown aetiology that can involve virtually any organ system. The disease is marked by prominent immune dysregulation, including the presence of diverse autoantibodies against nuclear antigens. Clinical presentation is highly variable, ranging from mild cutaneous and joint symptoms to severe, potentially life-threatening renal, haematological or neurological involvement.^1^ Besides, the risk of coronary artery disease is greatly increased in patients with SLE compared with an age-matched and sex-matched segment of the general population.^2^ Patients with SLE have an increased prevalence of traditional risk factors for cardiovascular (CV) disease, plus additional risk related to moderate to severe SLE, longstanding disease, lupus nephritis, antiphospholipid antibodies and glucocorticoid use.^3^ Patients with active SLE, particularly those with lupus nephritis and antiphospholipid syndrome, are at especially heightened CV risk. The underlying mechanisms of CV disease in SLE are complex and multifactorial. Chronic systemic inflammation and immune-mediated vascular injury are believed to contribute to accelerated atherosclerosis, a process characterised by immune activation, plaque development and increased risk of plaque rupture.^4 5^

Interleukin-2 (IL-2) is an essential cytokine predominantly produced by activated CD4+ T cells, with wide-ranging effects on the immune system.^6^ Acting as a T cell growth factor, IL-2 drives the proliferation of both CD4+ and CD8+ T cells and influences the development of cytotoxic and regulatory lymphocyte populations.^7^ It also enhances the cytolytic function and differentiation of natural killer (NK) cells, as well as the growth of B lymphocytes.^8^ In addition, IL-2 contributes to dendritic cell maturation and exerts both pro-inflammatory and anti-inflammatory effects, reflecting its complex regulatory role in immune homeostasis. In this regard, at low doses, IL-2 enhances regulatory T cells (Tregs), reducing inflammation, whereas at higher levels, it stimulates immune cells like T and NK cells, supporting defence against infections and tumours. With respect to this, IL-2 has been shown to inhibit growth of certain human tumour cells and decreased IL-2 production is often observed in the more advanced clinical stages of human tumours, which provides a rationale for inclusion of recombinant IL-2 in the immunotherapy for some tumours.^9^ High-dose bolus IL-2 was the first systemic immunotherapy shown to be effective in advanced melanoma. However, its clinical use has been limited by severe and potentially life-threatening CV, respiratory and infectious complications. Consequently, IL-2 therapy has largely been supplanted by the development of immune checkpoint inhibitors, which offer greater efficacy with reduced toxicity.^9 10^

To date, no large-scale studies have assessed serum IL-2 levels in patients with SLE. This is mainly because measuring IL-2 is technically challenging, as its serum concentrations are extremely low and undetectable with conventional methods. In the present study, we employed the highly sensitive Single Molecule Array (Simoa) technology, which allows quantification of IL-2 levels in serum at the femtogram-per-millilitre range. Our well-characterised SLE cohort underwent comprehensive evaluations of disease-specific parameters, including disease activity, autoantibody profiles and CV comorbidities. We further investigated the associations between serum IL-2 levels and disease activity, immunological features, lipid profile, metabolic syndrome-related parameters as well as carotid ultrasound findings as indicators of subclinical atherosclerosis and arterial stiffness.

## Materials and methods

### Study participants

This was a cross-sectional study that included 235 patients with SLE. All patients with SLE were 18 years or older, had a clinical diagnosis of SLE and met ≥4 American College of Rheumatology (ACR) classification criteria for SLE.^11^ Patients were recruited between July 2023 and July 2024. They had been diagnosed by rheumatologists and were regularly followed up in rheumatology outpatient clinics. Participation was allowed for patients taking prednisone, at an equivalent dose ≤10 mg/day, as glucocorticoids are often used in the treatment of SLE. The patient selection process is detailed in online supplemental figure 1. Of 312 records assessed, 67 were excluded (not meeting SLE criteria, age out of range, pregnancy/lactation, active infection/recent antibiotics, concomitant autoimmune/malignancy, or declined consent). A total of 245 patients consented and were screened; 10 were excluded postscreening due to missing/haemolysed serum or incomplete clinical/CV data. The final cohort included 235 patients with SLE. This study is reported as per the Strengthening the Reporting of Observational Studies in Epidemiology guideline (online supplemental table 1).

### Data collection

Individuals included in the study completed a CV risk factor and medication use questionnaire and underwent a physical examination. Weight, height, body mass index, abdominal circumference and systolic and diastolic blood pressure (measured with the participant in a supine position) were assessed under standardised conditions. Information regarding smoking status and hypertension treatment was obtained from the questionnaire. Medical records were reviewed to ascertain specific diagnoses and medications. SLE disease activity and damage were assessed using the SLE Disease Activity Index-2000 (SLEDAI-2K)^12^ and the Systemic Lupus International Collaborating Clinics (SLICC)/ACR Damage Index (SLICC-DI),^13^ respectively. For the present study proposal, the SLEDAI-2K index was divided into none (0 points), mild (1–5 points), moderate (6–10 points), high (11–19 points) and very high activity (>20 points) as described above, as previously described.^14^ Definitions of Remission in SLE (DORIS) was based on the absence of clinical disease activity as measured by the clinical SLEDAI-2K=0 and physician global assessment (PGA) <0.5. The patient may be on antimalarials, low-dose glucocorticoids (eg, prednisone ≤5 mg/day) and/or maintenance doses of immunosuppressive therapies.^15^ Similarly, Lupus Low Disease Activity State (LLDAS) accepts a SLEDAI-2K ≤4 with no activity from major organ systems, no new clinical activity compared with the previous assessment, a PGA of ≤1, prednisone dose ≤7.5 mg/day and maintenance doses of antimalarials and immunosuppressive therapies.^16^ The Systematic Coronary Risk Evaluation-2 (SCORE2) CV risk tool was calculated as previously described using age, gender, smoking status, systolic blood pressure and non-high-density lipoprotein (HDL)-cholesterol.^17^ SCORE2 estimates an individual’s 10-year risk of fatal and non-fatal CV disease events in individuals aged 40–69 years. For healthy people aged ≥ 70 years, the SCORE2-older persons algorithm estimates 5-year and 10-year fatal and non-fatal CV disease events.

### Laboratory assessments

Cholesterol, triglycerides and HDL-cholesterol were measured using the enzymatic colourimetric assay (Roche) in serum. Lipoproteins were assessed using a quantitative immunoturbidimetric assay (Roche) in serum. Cholesterol ranged from 0.08 to 20.7 mmol/L (intra-assay coefficient of variation of 0.3%); triglycerides ranged from 4 to 1.000 mg/dL (intra-assay coefficient of variation of 1.8%) and HDL-cholesterol ranged from 3 to 120 mg/dL (intra-assay coefficient of variation of 0.9%). The atherogenic index was calculated using the total cholesterol: HDL-cholesterol ratio according to the Castelli formula. Low-density lipoprotein (LDL)-cholesterol was calculated using the Friedewald formula. High-sensitivity C reactive protein (CRP) levels were measured using a high-sensitivity immunoassay.

The homeostatic model assessment (HOMA) method was performed to determine insulin resistance (IR). Briefly, the HOMA model enabled an estimate of insulin sensitivity (%S) and β-cell function (%B) from fasting plasma insulin, C-peptide and glucose concentrations. In this study, we used HOMA2, the updated computer HOMA model.^18^ This model can be used to assess insulin sensitivity and β-cell function from paired fasting plasma glucose and specific insulin, or C-peptide, concentrations across a range of 1–2200 pmol/L for insulin and 1–25 mmol/L for glucose. C-peptide better estimates β-cell function since it is a marker of secretion; and insulin data are preferable when calculating %S since HOMA-%S is derived from glucose disposal as a function of insulin concentration. In our study, IR and %S were calculated using insulin serum levels. Otherwise, %B was calculated using C-peptide serum levels. The computer model provided a value for insulin sensitivity expressed as HOMA2-%S (in which 100% is normal). HOMA2-IR (IR index) is simply the reciprocal of %S. Insulin (Architect Abbott, 2000I) and C-peptide (Immulite 2000, Siemens) were determined by chemiluminescent immunometric assays.

IL-2 and IL-6 levels were measured from serum samples through Simoa technique using the Cytokine 4-Plex C Advantage PLUS Reagent KitTM (no. 105066, Quanterix, Billerica, Massachusetts, USA) following the manufacturer’s instructions. Sample processing and analysis were done using an HD-X analyser (software V.4.16.2307.14001; Quanterix).

### Carotid ultrasound assessment

A carotid ultrasound examination was performed to evaluate the thickness of the carotid intima-media wall (cIMT) within the common carotid artery. The objective was to identify any localised plaques in the carotid arteries situated outside the skull (extracranial carotid tree). The measurements were carried out using the Esaote Mylab 70 ultrasound system from Genova, Italy. This system is equipped with a 7–12 MHz linear transducer and employs the Quality Intima Media Thickness in real-time automated software-guided radiofrequency technique developed by Esaote in Maastricht, the Netherlands. The assessment process adhered to the guidelines established in the Mannheim consensus,^19^ which establishes criteria for identifying plaques within the accessible extracranial carotid arteries. These arteries include the common carotid artery, the bulb and the internal carotid artery. Plaque criteria were established as the presence of a localised bulge within the arterial lumen, with a measurement of cIMT exceeding >1.5 mm. Additionally, the bulge needed to be at least 50% larger than the adjacent cIMT or result in an arterial lumen reduction of >0.5 mm.^19^

Carotid arterial stiffness was assessed using high-resolution ultrasound imaging with an Esaote system equipped with radiofrequency-based echo-tracking technology. The Esaote echo-tracking software automatically calculated key stiffness parameters, including pulse velocity wave, augmentation index, distensibility coefficient, compliance and the β-stiffness index, based on simultaneous recordings of arterial diameters and brachial blood pressure. All measurements were synchronised with the ECG to ensure accurate timing of systolic and diastolic phases. This standardised protocol allowed for the reliable and reproducible assessment of carotid stiffness, providing valuable insights into early vascular alterations associated with CV risk.^20^

### Statistical analysis

Demographic and clinical characteristics in patients with SLE were described as mean±SD or percentages for categorical variables. For non-normally distributed continuous variables, data were expressed as median and IQR. We based our sample size on detecting a correlation between IL-2 and SLEDAI-2K. Using Fisher’s z transformation with two-sided α=0.05 and 80% power, the required sample size is n≈3+((Zα/2+Zβ)/0.5·ln((1+ρ)/(1−ρ)))^2^. For an effect of ρ=0.20, this yields n≈193; for ρ=0.25, n≈123. Our final sample (n=235) provides ≥80% power to detect correlations as small as ~0.18–0.20. Missing data were addressed by carefully reviewing all collected variables for completeness prior to analysis. Cases with missing key clinical or laboratory data were excluded from specific analyses to avoid introducing bias. The extent of missing data was minimal and details of missing data are transparently reported. The relationship between disease characteristics and IL-2 serum levels was analysed using multivariable linear regression analysis adjusted for covariates associated with IL-2 at a significance level of p<0.20. Given the non-normal distribution of the IL-2 variable in the regression analyses, its log transformation was used. The results, expressed as beta-coefficients (β), should be interpreted according to this transformation. All the analyses used a 5% two-sided significance level and were performed using Stata software, V.17/SE (StataCorp, College Station, Texas, USA). P values <0.05 were considered statistically significant. Data visualisations were performed using Julius AI (Julius AI, San Francisco, California, USA; https://julius.ai), using Python 3.x.

## Results

### Demographics and disease-related data of patients with SLE

A comprehensive profile of the 235 patients with SLE included in this cohort is presented in table 1. The population was predominantly female (91%, n=214), with a mean age of 51±13 years. CV comorbidities were common, with 38% (n=90) having hypertension, 27% (n=64) classified as obese and 43% (n=100) diagnosed with metabolic syndrome. Smoking prevalence was 18% (n=42) and diabetes was present in 4% (n=10). The SCORE2 CV risk score had a median of 1.9% (IQR 1.0%–3.7%), with 84% (n=193) classified as low risk. Besides, carotid ultrasound measurements indicated a mean pulse wave velocity of 6.4±1.5 m/s, augmentation index median of 11% (IQR 2–29), mean carotid intima-media thickness of 681±124 microns and presence of carotid plaque in 42% of those assessed. Lipid profile and IR indices are also shown in table 1.

**Table 1:** Characteristics of patients with SLE and controls

	Patients with SLE (n=235)	Missing, n (%)
IL-2, fg/mL	131±88	0 (0)
Age, years	51±13	0 (0)
Female, n (%)	214 (91)	0 (0)
Body mass index, kg/m^2^	27±6	4 (2)
Abdominal circumference, cm	90±15	0 (0)
Waist circumference, cm	102±16	0 (0)
Waist-to-hip ratio	0.89±0.09	0 (0)
Cardiovascular comorbidity		
Smoking, n (%)	42 (18)	0 (0)
Diabetes, n (%)	10 (4)	0 (0)
Hypertension, n (%)	90 (38)	0 (0)
Obesity, n (%)	64 (27)	0 (0)
Metabolic syndrome, n (%)	100 (43)	1 (0)
Statins, n (%)	72 (31)	0 (0)
Aspirin, n (%)	53 (23)	0 (0)
SCORE2, %	1.9 (1.0–3.7)	0 (0)
SCORE2 categories		
Low risk	193 (84)	
Moderate risk	30 (13)	
High risk	6 (3)	
Carotid ultrasound		
Pulse wave velocity, m/s	6.4±1.5	10 (4)
Augmentation index, %	11 (2–29)	10 (4)
Carotid intima-media thickness, µm	681±124	10 (4)
Carotid plaque, n (%)	45 (42)	10 (4)
Laboratory data		
Cholesterol, mg/dL	178±38	1 (0)
Triglycerides, mg/dL	108±38	1 (0)
HDL-cholesterol, mg/dL	55±14	1 (0)
LDL-cholesterol, mg/dL	101±31	1 (0)
LDL:HDL-cholesterol ratio	1.93±0.72	1 (0)
Non-HDL-cholesterol, mg/dL	123±34	1 (0)
Lipoprotein A, mg/dL	30 (8–107)	1 (0)
Apo A1, mg/dL	157±27	1 (0)
Apo B, mg/dL	84±21	1 (0)
Apo B:Apo A1 ratio	0.55±0.15	1 (0)
Atherogenic index	3.37±0.92	1 (0)
Insulin resistance indices		
Glucose, mg/dL	90±17	1 (0)
Insulin, µU/mL	8.8 (5.6–15.9)	1 (0)
C-peptide, ng/mL	2.255±1.52	1 (0)
HOMA2-IR	1.0 (0.6–1.8)	1 (0)
HOMA2-%S	118±77	1 (0)
HOMA2-%B-C-peptide	149±63	1 (0)
SLE-related data		
Disease duration, years	19±12	2 (1)
CRP, mg/dL	1.6 (0.7–3.7)	1 (0)
IL-6, pg/mL	4.0 (2.4–7.4)	0 (0)
SLICC-DI	0 (1-0)	0 (0)
SLICC-DI ≥1, n (%)	90 (38)	0 (0)
SLEDAI-2K	0 (0–2)	0 (0)
SLEDAI categories, n (%)		
No activity, n (%)	122 (52)	
Mild, n (%)	99 (42)	
Moderate to high, n (%)	14 (6)	
DORIS, n (%)	177 (75)	0 (0)
LLDAS, n (%)	200 (85)	0 (0)
Auto-antibody profile		
Anti-DNA positive, n (%)	171 (73)	0 (0)
Anti-ENA positive, n (%)	157 (67)	0 (0)
Anti-SSA, n (%)	74 (31)	0 (0)
Anti-SSB, n (%)	26 (11)	0 (0)
Anti-RNP, n (%)	51 (22)	0 (0)
Anti-Sm, n (%)	33 (14)	0 (0)
Antiribosome	22 (9)	0 (0)
Antinucleosome	42 (18)	0 (0)
Antihistone	30 (13)	0 (0)
Antiphospholipid syndrome, n (%)	34 (14)	0 (0)
Antiphospholipid autoantibodies, n (%)	74 (31)	1 (0)
Lupus anticoagulant, n (%)	54 (23)	1 (0)
ACA IgM, n (%)	30 (13)	1 (0)
ACA IgG, n (%)	40 (17)	1 (0)
Anti-β2 glycoprotein IgM, n (%)	20 (9)	1 (0)
Anti-β2 glycoprotein IgG, n (%)	25 (11)	1 (0)
Current prednisone, n (%)	65 (28)	0 (0)
Prednisone, mg/day	5 (2.5–5)	0 (0)
Hydroxychloroquine, n (%)	173 (74)	0 (0)
Methotrexate, n (%)	26 (11)	0 (0)
Mycophenolate mofetil, n (%)	30 (13)	0 (0)
Azathioprine, n (%)	16 (7)	0 (0)
Anifrolumab, n (%)	3 (1)	0 (0)
Rituximab, n (%)	6 (3)	0 (0)
Belimumab, n (%)	29 (12)	0 (0)

Data represent means±SD or median (IQR) when data were not normally distributed.

SLEDAI categories were defined as: 0, no activity; 1–5 mild; 6–10 moderate; >10 high activity, >20 very high activity.

ACA, anticardiolipin; Anti-RNP, anti-ribonucleoprotein; Anti-Sm, anti-Smith; Anti-SSA, anti-Sjögren’s syndrome antigen A; Anti-SSB, anti-Sjögren’s syndrome antigen B; Apo, apolipoprotein; %B, β-cell function; C3 C4, complement; CRP, C reactive protein; DMARD, disease-modifying antirheumatic drug; DORIS, Definitions of Remission in SLE; ENA, extractable nuclear antibodies; HDL, high-density lipoprotein; HOMA-IR, homeostatic model assessment of insulin resistance; IL, interleukin; LDL, low-density lipoprotein; LLDAS, Lupus Low Disease Activity State; %S, insulin sensitivity; SCORE2, Systematic Coronary Risk Evaluation-2; SLEDAI-2K, SLE Disease Activity Index-2000; SLICC-DI, Systemic Lupus International Collaborating Clinics/American Colleague of Rheumatology Damage Index.

The average disease duration was 19±12 years. Disease activity measures reported a median SLEDAI-2K score of 0 (IQR 0–2), with over half (52%, n=122) classified as having no active disease and 79% (n=184) in remission according to DORIS criteria. Organ damage assessed by SLICC-DI showed a median of 0 (IQR 1–0) and 38% had a score ≥1. Autoantibody profiles were consistent with classic SLE features: 73% anti-DNA positive, 67% anti-extractable nuclear antibody positive and 31% anti-Sjögren’s syndrome antigen A (SSA) positive. Antiphospholipid syndrome was diagnosed in 14% (n=34), with 31% (n=74) expressing antiphospholipid autoantibodies. Common treatments included hydroxychloroquine (74%, n=173), prednisone (28%, n=65) with a median dose of 5 mg/day (IQR 2.5–5) and immunosuppressants like mycophenolate mofetil (13%, n=30) and methotrexate (11%, n=26). Biologic therapies such as anifrolumab and belimumab were less frequent (1% and 12%, respectively) (table 1).

### Disease-related data association with IL-2 serum values

Table 2 presents the associations between various disease-related variables and serum IL-2 levels (log transformed), analysed as the dependent variable. Both univariable and multivariable linear regression analyses were performed, with the multivariable model adjusted for smoking and statin use. No significant associations with serum IL-2 levels were observed for demographic factors such as age, sex, body mass index or anthropometric measurements. Among CV comorbidities, smoking showed a borderline negative association with IL-2 (p=0.051).

**Table 2:** Relationship of serum IL-2 with clinical and immunological features in SLE

	Log IL-2, fg/mL			
	Beta-coefficient (95% CI), p value			
	Univariable		Multivariable	
Age, years	0.003 (−0.003 to 0.01)	0.33		
Female	0.05 (−0.21 to 0.30)	0.72		
Body mass index, kg/m^2^	0.01 (−0.01 to 0.02)	0.37		
Abdominal circumference, cm	0.002 (−0.003 to 0.01)	0.48		
Waist circumference, cm	0.002 (−0.002 to 0.01)	0.31		
Waist-to-hip ratio	−0.07 (−0.87 to 0.73)	0.85		
Cardiovascular comorbidity				
Smoking	−0.19 (−0.38 to 0.001)	0.052		
Diabetes	0.08 (−0.28 to 0.45)	0.65		
Hypertension	0.08 (−0.07 to 0.23)	0.30		
Obesity	0.10 (−0.06 to 0.27)	0.21		
Statins	0.13 (−0.03 to 0.29)	0.11		
Aspirin	0.06 (−0.11 to 0.24)	0.49		
SLE-related data				
Disease duration, years	0.003 (−0.003 to 0.01)	0.37		
CRP, mg/dL	**0.02 (0.001 to 0.04**)	**0.036**	**0.02 (0.003 to 0.04**)	**0.021**
IL-6, pg/mL	**0.01 (0.01 to 0.02**)	**<0.001**	**0.01 (0.006 to 0.02**)	**<0.001**
SLICC-DI	0.05 (−0.02 to 0.12)	0.19	0.04 (−0.03 to 0.12)	0.24
SLICC-DI ≥1	0.09 (−0.06 to 0.24)	0.25		
SLEDAI-2K	**0.03 (0.001 to 0.07**)	**0.043**	**0.04 (0.002 to 0.07**)	**0.040**
SLEDAI categories				
No activity	ref.		ref.	
Mild	0.06 (−0.09 to 0.21)	0.41	0.05 (−0.10 to 0.20)	0.52
Moderate to high	**0.34 (0.02 to 0.65**)	**0.036**	**0.34 (0.03 to 0.65**)	**0.033**
DORIS	−0.12 (−0.29 to 0.05)	0.16	−0.11 (−0.28 to 0.06)	0.21
LLDAS	−0.09 (−0.30 to 0.11)	0.38		
Auto-antibody profile				
Anti-DNA positive	**0.24 (0.08 to 0.40**)	**0.003**	**0.23 (0.07 to 0.39**)	**0.006**
Anti-ENA positive	**0.19 (0.04 to 0.35**)	**0.015**	**0.18 (0.03 to 0.34**)	**0.019**
Anti-SSA	**0.22 (0.07 to 0.38**)	**0.005**	**0.24 (0.09 to 0.40**)	**0.002**
Anti-SSB	**0.23 (0.001 to 0.46**)	**0.049**	**0.23 (0.001 to 0.46**)	**0.049**
Anti-RNP	0.11 (−0.07 to 0.28)	0.24		
Anti-Sm	**0.31 (0.10 to 0.52**)	**0.004**	**0.30 (0.10 to 0.51**)	**0.004**
Antiribosome	**0.29 (0.04 to 0.54**)	**0.004**	**0.29 (−0.05 to 0.54**)	**0.020**
Antinucleosome	0.09 (−0.10 to 0.29)	0.33		
Antihistone	0.16 (−0.06 to 0.38)	0.15	0.18 (−0.04 to 0.40)	0.11
Antiphospholipid syndrome	0.02 (−0.19 to 0.23)	0.85		
Antiphospholipid autoantibodies	0.03 (−0.13 to 0.19)	0.69		
Lupus anticoagulant	−0.02 (−0.19 to 0.16)	0.87		
ACA IgM	0.03 (−0.19 to 0.25)	0.81		
ACA IgG	0.07 (−0.12 to 0.27)	0.47		
Anti-β2 glycoprotein IgM	0.15 (−0.11 to 0.41)	0.27		
Anti-β2 glycoprotein IgG	0.17 (−0.07 to 0.41)	0.16	0.17 (−0.07 to 0.41)	0.16
Current prednisone	0.15 (−0.01 to 0.31)	0.070	0.13 (−0.03 to 0.29)	0.12
Prednisone, mg/day	−0.03 (−0.08 to 0.01)	0.16		
Hydroxychloroquine	0.002 (−0.16 to 0.17)	0.98		
Methotrexate	0.13 (−0.10 to 0.36)	0.27		
Mycophenolate mofetil	0.09 (−0.13 to 0.31)	0.41		
Azathioprine	0.04 (−0.25 to 0.33)	0.79		
Anifrolumab	0.02 (−0.63 to 0.67)	0.95		
Rituximab	−0.30 (−0.76 to 0.17)	0.21		
Belimumab	0.06 (−0.16 to 0.28)	0.58		

In this analysis, IL-2 serum values are the dependent variable. Multivariable analysis is adjusted for smoking and statin use.

Significant p values are depicted in bold.

SLEDAI categories were defined as: 0, no activity; 1–5 mild; 6–10 moderate; >10 high activity, >20 very high activity.

ACA, anticardiolipin; Anti-RNP, anti-ribonucleoprotein; Anti-Sm, anti-Smith; Anti-SSA, anti-Sjögren’s syndrome antigen A; Anti-SSB, anti-Sjögren’s syndrome antigen B; CRP, C reactive protein; DORIS, Definitions of Remission in SLE; ENA, extractable nuclear antibodies; IL, interleukin; LLDAS, Lupus Low Disease Activity State; ref., reference; SLEDAI-2K, SLE Disease Activity Index 2000; SLICC-DI, Systemic Lupus International Collaborating Clinics/American Colleague of Rheumatology Damage Index.

**Figure 1:**
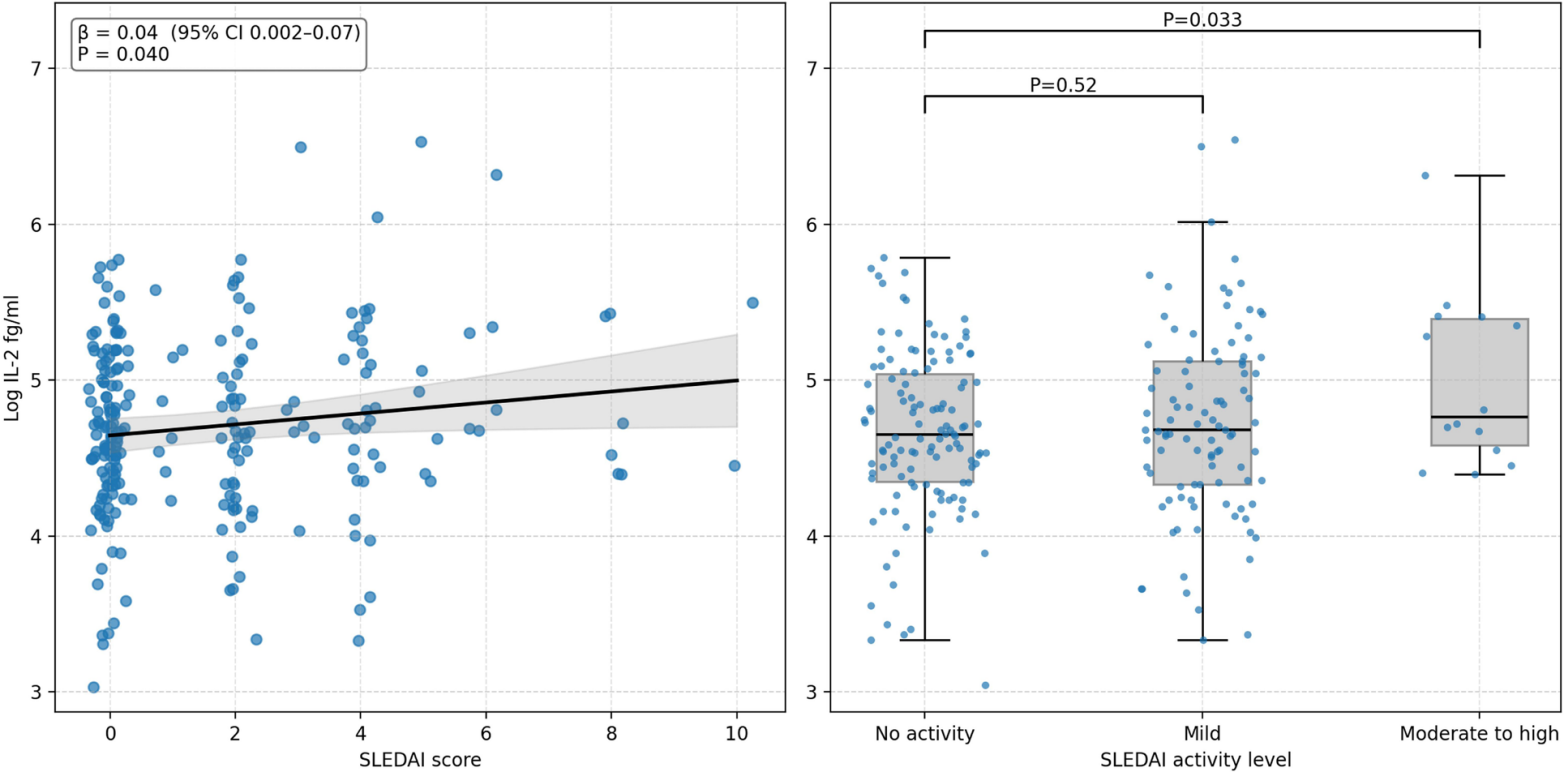
Association between SLE Disease Activity Index (SLEDAI) score and interleukin 2 (IL-2) levels adjusted for smoking and statin use. Left panel: scatter plot showing the relationship between SLEDAI score (x-axis) and log-IL-2 levels in fg/mL (y-axis). The black line represents the adjusted linear regression line with 95% CI (grey shaded area). The regression was adjusted for smoking status and statin use. Beta-coefficient=0.04 (95% CI 0.002 to 0.07), p=0.040. Each point represents an individual patient with slight horizontal jittering applied for visualisation purposes. Right panel: box plots comparing log-IL-2 levels (adjusted for smoking and statin use) across SLEDAI score categories. Individual data points are overlaid on the box plots with horizontal jittering. Box plots show median (black line), IQR (box) and whiskers extending to 1.5 times the IQR. Statistical comparisons between categories are shown: category mild versus no activity, p=0.52; category moderate to high versus no activity, p=0.033. Log-IL-2 values were adjusted by removing the effects of smoking and statin use while preserving the overall mean.

With respect to disease-related variables, elevated serum IL-2 levels were significantly associated with markers of inflammation, including CRP (β=0.02, 95% CI 0.003 to 0.04, p=0.021) and IL-6 (β=0.01, 95% CI 0.006 to 0.02, p<0.001). Disease activity, assessed by SLEDAI-2K, also showed a positive association with IL-2 levels (β=0.04, 95% CI 0.002 to 0.07, p=0.040), an effect that was particularly pronounced in patients with moderate to high disease activity (β=0.34, 95% CI 0.03 to 0.65, p=0.033) (figure 1). Further analysis of individual SLEDAI items in relation to serum IL-2 levels revealed that anti-DNA positivity and leucopenia were the only components significantly associated with IL-2 elevation, whereas renal (proteinuria), cutaneous, neurological and articular organ involvement showed no significant relationship (online supplemental table 1). Autoantibody profile revealed significant positive associations between IL-2 and anti-DNA, anti-SSA, anti-Sjögren’s syndrome antigen B (SSB), anti-Smith (Sm) and antiribosome. Regarding current treatments, immunosuppressive treatments, including hydroxychloroquine, methotrexate, mycophenolate mofetil, azathioprine and biologics, showed no statistically significant associations (table 2).

### Analysis of cardiovascular disease-related factors associated with IL-2

The multivariable analysis assessing the association between CV disease-related factors and serum IL-2 levels (log transformed) in patients with SLE is shown in table 3. The analysis was adjusted for smoking and statin use. Among lipid profile parameters, triglyceride levels showed a positive association with serum IL-2 in the multivariable analyses (β=0.0018, 95% CI 0.0006 to 0.003, p=0.004). Besides, HDL-cholesterol exhibited a significant inverse association with IL-2 (β=−0.0061, 95% CI −0.01 to −0.0009, p=0.022) after adjustment for covariates. Also, a non-significant trend towards a negative association was observed for apolipoprotein A1 and lipoprotein(a), and towards a positive relation to the atherogenic index, although statistical significance was not reached in these cases (table 3). Remarkably, when this analysis was replicated in patients not taking statins, the relationship between triglycerides and IL-2 persisted significantly (data not shown).

**Table 3:** Associations between serum IL-2 and cardiovascular risk factors

	Log IL-2, fg/mL			
	Beta-coefficient (95% CI), p value			
	Univariable		Multivariable	
Lipid profile				
Cholesterol, mg/dL	−0.001 (−0.003 to 0.0008)	0.24	**0.0018 (0.0006 to 0.0030**)	**0.004**
Triglycerides, mg/dL	**0.002 (0.0007 to 0.003**)	**0.002**	**−0.0061 (−0.01 to −0.0009**)	**0.022**
HDL-cholesterol, mg/dL	**−0.006 (−0.01 to −0.0006)**	**0.029**	−0.0015 (−0.0038 to 0.0009)	0.23
LDL-cholesterol, mg/dL	−0.002 (−0.004 to 0.0004)	0.11		
LDL:HDL-cholesterol ratio	0.02 (−0.09 to 0.1)	0.76		
Non-HDL-cholesterol, mg/dL	−0.0004 (−0.003 to 0.002)	0.69	−0.0008 (−0.0017 to 0.0000)	0.062
Apo A1, mg/dL	−0.0008 (−0.002 to 0.0001)	0.073	−0.0026 (−0.0054 to 0.0001)	0.057
Apo B, mg/dL	−0.002 (−0.005 to 0.0004)	0.094		
Apo B:Apo A1 ratio	−0.001 (−0.005 to 0.002)	0.50		
Atherogenic index	0.1 (−0.4 to 0.6)	0.64		
Insulin resistance indices				
Metabolic syndrome	0.14 (−0.02 to 0.29)	0.055	−0.0025 (−0.0069 to 0.0020)	0.28
Glucose, mg/dL	−0.002 (−0.006 to 0.003)	0.46		
Insulin, µU/mL	−0.001 (−0.006 to 0.004)	0.70		
C-peptide, ng/mL	0.05 (−0.002 to 0.09)	0.058	0.04 (−0.01 to 0.09)	0.12
HOMA2-IR	0.01 (−0.06 to 0.04)	0.68		
HOMA2-%S	−0.0004 (−0.0013 to 0.0006)	0.47		
HOMA2-%B-C-peptide	**0.001 (0.0002 to 0.003**)	**0.018**	**0.0013 (0.0001 to 0.0024**)	**0.032**
Carotid ultrasound				
Pulse wave velocity, m/s	0.02 (−0.05 to 0.10)	0.57		
Augmentation index, %	−0.03 (−0.01 to 0.005)	0.44		
Carotid intima-media thickness, mm	0.31 (−0.50 to 1.12)	0.45		
Carotid plaque, n (%)	−0.006 (−0.21 to 0.19)	0.95		
SCORE2 calculator	−0.002 (−0.02 to 0.02)	0.89		
Low risk	ref.			
Moderate risk	−0.10 (−0.33 to 0.12)	0.36		
High risk	0.03 (−0.44 to 0.50)	0.89		

In this analysis, serum levels of IL-2 are the dependent variable. Multivariable analysis is adjusted for smoking and statin use.

Significant p values are depicted in bold.

Apo, apolipoprotein; HDL, high-density lipoprotein; HOMA2-%B-C peptide, β-cell function index through homeostatic model assessment (calculated with glucose and C peptide serum levels); HOMA2-IR, insulin resistance index through homeostatic model assessment (calculated with glucose and insulin serum levels); HOMA2-%S, insulin sensitivity index through homeostatic model assessment (calculated with glucose and insulin serum levels); IL, interleukin; LDL, low-density lipoprotein; ref., reference; SCORE2, Systematic Coronary Risk Evaluation-2.

The presence of metabolic syndrome was not significantly linked to IL-2 levels. However, serum IL-2 levels showed a significant positive association with β-cell dysfunction (β=0.0013, 95% CI 0.0001 to 0.0024, p=0.032). Contrary to this, carotid ultrasound parameters including pulse wave velocity, augmentation index, carotid intima-media thickness and presence of carotid plaque were not associated with IL-2 levels. Finally, CV risk calculated by the SCORE2 tool did not correlate with IL-2 levels either, across low-risk, moderate-risk and high-risk categories (table 3).

## Discussion

To our knowledge, this is the first study to evaluate serum IL-2 levels in a large and well-characterised cohort of patients with SLE. Our findings demonstrate that IL-2 is associated with markers of systemic inflammation, disease activity and autoantibody profiles, supporting its potential involvement in SLE pathogenesis. In addition to this immunological role, IL-2 levels were also linked to an altered lipid profile and beta-cell dysfunction, a metabolic disturbance frequently reported in SLE, suggesting that IL-2 may contribute to autoimmune mechanisms and to cardiometabolic pathways that increase CV risk.

The critical role of IL-2 in immune homeostasis has been established in early studies showing that the deletion of the IL-2 gene in mice resulted in the development of autoimmune disease, in particular ulcerative colitis.^21^ Autoimmune manifestations were also observed in mice deficient of the genes that encode for the IL-2 receptor.^22^ Besides, in lupus-prone mice, the numbers of Treg cells, which critically depend on IL-2, gradually decline as the disease progresses.^23^ Historically, serum IL-2 levels in patients with SLE were reported to be lower than in healthy controls, suggesting that IL-2 deficiency may contribute to disease onset and progression.^24^ However, a recent report confirmed that the levels of serum soluble form of interleukin-2 receptor (sIL-2Rα) were increased significantly in patients with SLE and closely related to the disease activity of patients with SLE.^25^ This study showed that in the peripheral blood of patients with SLE, sIL-2Rα can bind IL-2, reducing its measurable concentration and biological activity, thereby suppressing Treg differentiation and compromising peripheral tolerance.^25^ In our study, we observed a positive association between serum IL-2 levels, disease activity and ANA presence. This difference may likely reflect our use of an ultrasensitive and highly reliable assay, capable of detecting subtle variations and intrapatient heterogeneity that conventional methods miss. Additionally, elevated sIL-2Rα can mask true IL-2 concentrations when measured with less sensitive assays.

At first glance, it seems paradoxical that IL-2 levels can be positively associated with disease activity in SLE. However, this apparent contradiction can be explained by several immunological mechanisms. In this regard, in active disease, the immune system is highly stimulated, leading to transient IL-2 production by activated T cells and other immune cells as a compensatory response to inflammation and tissue damage. However, this increase may not necessarily reflect a functional or beneficial IL-2 response, as it may be insufficient to restore immune regulation. Additionally, IL-2 is not exclusively produced by Tregs as effector and autoreactive T cells can also secrete IL-2 during activation, potentially contributing to disease pathology. Moreover, SLE is associated with defects in IL-2 receptor signalling and consumption, meaning that even when IL-2 is present in the serum, it may not be effectively used by Tregs or other regulatory mechanisms, resulting in an apparent accumulation. Therefore, as discussed above, our results may indicate that, when measured with ultrasensitive techniques, IL-2 levels correlate positively with disease activity and autoantibody presence, reflecting dynamic changes in the inflammatory environment of SLE.

Low-dose IL-2 has been investigated as a therapeutic approach for disease control in patients with SLE.^25 26^ Evidence for its therapeutic benefit in SLE first emerged from a case report of a patient aged 36 years with marked clinical and serological improvement after IL-2 therapy,^27^ followed by a small series of five patients with refractory SLE in whom daily low-dose IL-2 selectively expanded Treg despite the absence of formal disease activity assessment.^28^ Subsequent larger studies have strengthened this evidence. An open-label trial in 50 patients with refractory SLE who received intermittent low-dose IL-2 combined with rapamycin demonstrated significant improvements in disease activity and in the T helper 17 (Th17)/Treg balance over 24 weeks.^29^ Two clinical trials further confirmed these findings: an open-label study in 38 patients with active SLE reported >89% SLE Responder Index-4 (SRI-4) response at week 12 with concomitant glucocorticoid sparing,^30^ and a randomised placebo-controlled trial in 60 patients showed higher SRI-4 in the IL-2 group compared with placebo at week 24, as well as higher complete remission rates of lupus nephritis.^31^ Importantly, both trials reported good safety profiles and improvements in Treg function, with additional effects on NK cells and reduction in the TFH+Th17/Treg ratio.^30 31^ Currently, two phase II trials are ongoing to further assess the efficacy and safety of low-dose IL-2 in SLE.^26^ Although our findings demonstrate a positive association between serum IL-2 levels and both disease activity and the autoimmune profile in SLE, this association does not preclude a potential beneficial role of exogenous low-dose IL-2 therapy. This apparent paradox likely reflects the dual and context-dependent role of IL-2: while higher endogenous levels may be linked to inflammatory activity, controlled administration at low doses could have therapeutic potential by correcting immune dysregulation in SLE.

In our study, we observed a significant association between serum IL-2 levels and disease activity as measured by the SLEDAI score, while no correlation was found with the SLICC-DI, which reflects accumulated organ damage. This differential association may indicate that IL-2 is predominantly involved in the acute inflammatory phases of SLE rather than in chronic or irreversible damage processes. Supporting this notion, although not reaching statistical significance, patients in low disease activity such as LLDAS or clinical remission by DORIS criteria exhibited lower IL-2 levels. Notably, SLEDAI items that correlated with IL-2 levels were primarily those reflecting anti-DNA positivity and leucopenia, whereas items denoting active organ damage showed no significant association. However, it should be noted that the prevalence of active organ involvement was low in this cohort, which may have limited the power to detect significant correlations. Collectively, these findings suggest that IL-2 in SLE primarily reflects active inflammation rather than chronic damage, highlighting its role as a biomarker of disease activity in the dynamic inflammatory milieu of the disease. Moreover, in the multivariable analysis, we observed an association of IL-2 with CRP and IL-6. Therefore, in active disease, increased IL-2 levels in serum can be a marker of immune dysregulation, not a sign of functional or beneficial immune activity.

IL-2 has been described to also play a crucial protective role in maintaining vascular homeostasis and preventing atherosclerosis.^32^ The Low-dose InterLeukin-2 in Patients with Stable Ischaemic Heart Disease and Acute Coronary Syndromes study demonstrated the safety and biological efficacy of low-dose IL-2 in individuals with ischaemic heart disease.^33^ This has led to the initiation of the IVORY trial, designed to assess the effects of low-dose IL-2 on vascular inflammation in patients with acute coronary syndromes. This randomised, placebo-controlled phase II study has reported preliminary data showing a 7.7% reduction in arterial inflammation with a positive safety profile and indicated a potential reduction in major adverse CV events. In our study, we observed a positive association between serum IL-2 levels and lipid profile, suggesting that elevated IL-2 concentrations may be linked to the inflammatory dyslipidaemia that frequently accompanies SLE. While low-dose IL-2 therapy has demonstrated cardioprotective properties, as outlined above, our findings suggest that higher endogenous IL-2 levels within the inflammatory milieu may have a CV risk-promoting effect. This dual role reflects the dose-dependent and context-dependent nature of IL-2: at elevated levels, it may promote immune cell activation and contribute to vascular inflammation and atherosclerosis, whereas at low, controlled doses, it selectively expands Tregs and supports CV homeostasis. Altogether, these results highlight the complex role of IL-2 in CV disease and underscore the importance of distinguishing between its physiological and therapeutic effects in CV disease.

IL-2 has been previously linked to IR. In a previous report, subcutaneous fat tissue samples were collected from 56 healthy individuals, and IL-2, along with other inflammatory mediators, was quantified using quantitative reverse transcription-PCR and immunohistochemistry.^34^ Notably, IL-2 was associated positively with fasting blood glucose, HbA1c, triglycerides and CRP. The authors of that study concluded that increased expression of IL-2 in adipose tissue, particularly in the context of obesity, may represent a novel biomarker for the progression of metabolic inflammation and IR. In our study, we identified a positive significant association between serum IL-2 levels and β-cell dysfunction, as assessed by HOMA2-%B. This finding suggests that in patients with SLE, β-cell impairment could be, at least in its early stages, influenced or mediated by IL-2. Such an association supports the notion that immune dysregulation, particularly involving IL-2 pathways, could contribute to metabolic disturbances observed in SLE, linking immune activation with altered β-cell function.

The measurement of IL-2 in this study was performed using the highly sensitive Simoa platform, which surpasses conventional assays by allowing detection of extremely low serum IL-2 concentrations with high specificity and reproducibility. Nevertheless, some limitations should be noted. First, the cross-sectional design does not permit inferences about causality or the temporal sequence between IL-2 levels and clinical features. Second, the absence of a healthy control group prevents direct comparison of IL-2 concentrations between patients with SLE and the general population. However, it is important to emphasise that the main objective of our work was to explore the relationships between serum IL-2 levels and clinical and immunological characteristics within the SLE cohort.

In conclusion, our study demonstrates that serum IL-2 levels in patients with SLE are positively associated with markers of systemic inflammation, disease activity, specific autoantibody profiles and certain lipid abnormalities. These findings support a role for IL-2 as a biomarker reflecting active inflammatory and immunological processes in SLE. Besides, our results highlight the complex and multifaceted role of IL-2 in SLE pathogenesis and its potential utility as a target for immunomodulatory therapies.

## Supplementary material

10.1136/lupus-2025-001870 online supplemental figure 1

10.1136/lupus-2025-001870 online supplemental table 1

## Data Availability

Data are available on reasonable request.
