# Supplementary material for: Serum IL-2 levels are associated with disease activity and related to dyslipidaemia and the immunological profile in systemic lupus erythematosus
Source: Lupus Sci Med. 2026 Mar 12;13(1):e001870. doi: 10.1136/lupus-2025-001870 (PMC12983779; doi:10.1136/lupus-2025-001870)
Supplement: online supplemental figure 1 [file lupus-13-1-s001.docx]

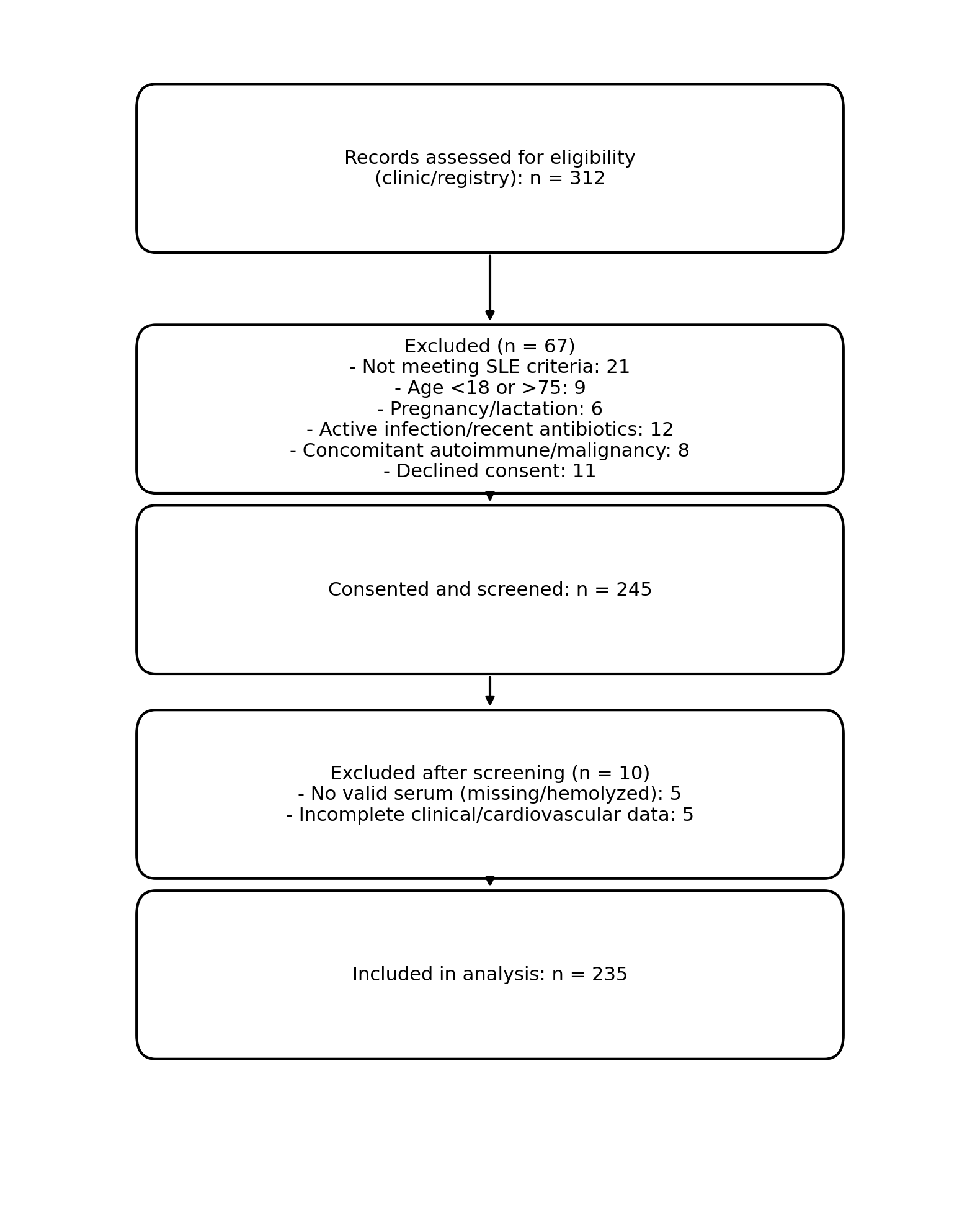


**Supplementary Figure 1**. Study flow diagram depicting patient screening, eligibility, and inclusion. Of 312 records assessed, 67 were excluded (not meeting SLE criteria, age out of range, pregnancy/lactation, active infection/recent antibiotics, concomitant autoimmune/malignancy, or declined consent). A total of 245 patients consented and were screened; 10 were excluded post-screening due to missing/hemolyzed serum or incomplete clinical/cardiovascular data. The final cohort included 235 SLE patients analyzed for associations between serum IL‑2 and clinical/cardiovascular features.
