# Supplementary material for: Serum IL-2 levels are associated with disease activity and related to dyslipidaemia and the immunological profile in systemic lupus erythematosus
Source: Lupus Sci Med. 2026 Mar 12;13(1):e001870. doi: 10.1136/lupus-2025-001870 (PMC12983779; doi:10.1136/lupus-2025-001870)
Supplement: online supplemental table 1 [file lupus-13-1-s002.docx]

| **Supplementary Table 1. SLEDAI-2k items relation to IL-2 serum values** | | | | |
| --- | --- | --- | --- | --- |
|  |  |  | *log* IL2, fg/ml | |
|  | n | % | beta coef. (95%CI) | p |
| Seizures | 0 | 0 | - |  |
| Psychosis | 1 | 0 | 0.09 (-0.06-0.23) | 0.22 |
| Organic brain syndrome | 0 | 0 | - |  |
| Visual disturbance | 0 | 0 | - |  |
| Cranial nerve disorder | 0 | 0 | - |  |
| Lupus headache | 0 | 0 | - |  |
| ACVA | 0 | 0 | - |  |
| Vasculitis | 0 | 0 | - |  |
| Arthritis | 10 | 4 | -0.04 (-0.13-0.06) | 0.45 |
| Myositis | 0 | 0 | - |  |
| Urinary cylinders | 0 | 0 | - |  |
| Hematuria | 1 | 0 | -0.08 (-0.36-0.20) | 0.58 |
| Proteinuria | 7 | 3 | 0.06 (-0.05-0.16) | 0.31 |
| Pyuria | 2 | 1 | 0.03 (-0.17-0.23) | 0.78 |
| Rash | 13 | 6 | 0.05 (-0.11-0.21) | 0.53 |
| Alopecia | 5 | 2 | 0.06 (-0.19-0.32) | 0.63 |
| Mucosal ulcers | 11 | 5 | 0.14 (-0.04-0.31) | 0.12 |
| Pleurisy | 1 | 0 | **-0.67 (-1.23-(-0.12))** | **0.018** |
| Pericarditis | 0 | 0 | - |  |
| Low complement | 55 | 23 | 0.01 (-0.08-0.09) | 0.90 |
| Elevated anti-DNA | 171 | 73 | **0.24 (0.08–0.40)** | **0.003** |
| Fever | 1 | 0 | -0.30 (-1.43-0.82) | 0.60 |
| Thrombopenia | 24 | 10 | 0.12 (-0.12-0.36) | 0.33 |
| Leukopenia | 48 | 21 | **0.39 (0.21-0.56)** | **<0.001** |
| ACVA: Acute Cerebrovascular Accident. | | | |  |
| Significant b values are depicted in bold. | | | |  |
